# The efficacy of 8% Arginine-CaCO3 applications on dentine hypersensitivity following periodontal therapy: A clinical and scanning electron microscopic study

**DOI:** 10.4317/medoral.17990

**Published:** 2012-12-10

**Authors:** Ahu Uraz, Özge Erol-Şii?mşek, Selcen Pehlii?van, Zekiye Suludere, Belgin Bal

**Affiliations:** 1 PhD, Assist. Prof, Department of Periodontology, Faculty of Dentistry, Gazi University, Ankara, Turkey; 2 DDS, Department of Periodontology, Faculty of Dentistry, Gazi University, Ankara, Turkey; 3 MA, Department of Biostatistics, Faculty of Medicine, Ankara University, Ankara, Turkey; 4 PhD, Prof, Department of Zoology and Botany (Biology), Faculty of Science, Gazi University, Ankara, Turkey; 5 PhD, Prof, Department of Periodontology, Faculty of Dentistry, Gazi University, Ankara, Turkey

## Abstract

Objectives: Periodontal therapy is one of the etiological factors of dentine hypersensitivity (DH). This study aimed to evaluate the efficacy of %8Arginine-CaCO3 on DH that affects patients after periodontal treatment. Study design: Seventy-one teeth from the volunteers (n=36) with history of DH caused by periodontal therapy were included in this study, and randomly divided into two groups: group-1, who received 8%Arginine-CaCO3 and group-2, who received 1.23%NaF-gel. The clinical indices were recorded at first visit.DH was evaluated by using tactile, air-blast, and thermal stimuli. The subject’s response was recorded at baseline, immediately (Day-0) and one month after the application. Results and conclusions: The results were statistically analyzed, and it was found that 8% Arginine-CaCO3 treatment was more effective than 1.23% NaF-gel at time intervals. Sensitivity score differences between the groups were statistically significant at Day-28. The 8% Arginine-CaCO3 group exhibited statistically significant reduction in DH on three stimuli at baseline to Day-28. It was concluded that 8% Arginine-CaCO3 is more effective than 1.23% NaF-gel in reduction of patients’ pain.

** Key words:**Arginine, desensitizing agent, hypersensitivity, periodontal treatment, scaling and root planning, sodium fluoride.

## Introduction

Dentine hypersensitivity (DH) is characteristically in response to an array of stimuli which is thermal, tactile, evaporative, osmotic or chemical and which cannot be attributed to any other form of dental defect, disease or pathology (1-3). The exposed dentin can be affected from stimuli due to loss of enamel or cementum (4). DH is typically common clinical condition, and previous studies have suggested higher prevalence levels up to 57%. The emergence of sensitivity to even touch the root dentin surface lightly with a tooth brush causes deficiency of oral hygiene practices (5). There are varied etiologic and predisposing factors related to DH including acute and chronic inflammatory periodontal diseases, trauma, tooth flexure, occlusal forces, attrition, abrasion, erosion, tooth brushing, parafunctional habits, periodontal treatment, and acidic dietary components (1,6-8).

One of the important components of periodontal treatment is mechanical debridement. Non surgical therapy including scaling and root planning is the most commonly used procedure. It designed to remove dental deposits and necrotic cementum from the root surface. The emergence of dentin hypersensitivity after the periodontal treatment is expected 

result. Dentin hypersensitivity is most prevalent in the cervical area of the roots, where the cementum is very thin. Periodontal procedures may entirely remove this thin cementum layer and induce hypersensitivity or, more correctly, root hypersensitivity. The prevalence of sensitivity observed to increase after scaling and root planning, compared to baseline was reported (4,9).

Several theories have been proposed to explain the mechanism of DH. Scientific evidence supports the hydrodynamic theory. The main initial symptom of hypersensitivity is sharp, sudden pain and disappears when the stimulus is removed. The fact that root surfaces become sensitive to a variety of external stimuli after periodontal instrumentation and dentinal tubules become uncovered to the oral environment and to hydrodynamic forces (1,6,9). When thermal and osmotic stimuli is applied to dentine, liquid in the dentin tubules are replaced rapidly. The move may stimulate nerve fibers of pulp mechanically, thereby inducing a painful sensation according to the hydrodynamic theory of dentin sensitivity (1,9-11). This mechanism can certainly explain the sensitivity that patients experience immediately after the instrumentation procedure and during a short period afterwards, while it does not make clear why the symptoms increase over time and why the pain condition may affect certain patients and certain teeth (2,3,12).

Most treatment procedures used commonly are based on desensitizing agents, which the proposed mode of action is tubule occlusion and/or desensitization (1,3). In the treatment of dentine hypersensitivity, variety of methods and materials are applicable to both home and office use (1,2,4,12,13). The agents used at home include desensitizing dentifrices or mouthwashes with sodium fluoride (NaF), stannous fluoride, potassium nitrate, formaldehyde, strontium chloride, etc. The agents used at office include fluoride compounds, calcium compounds, cavity varnishes, restorative resins, etc. (4,7,9,11,13). Previous clinical studies have shown that treatment of exposed root surfaces with fluoride toothpaste and concentrated fluoride solutions is very efficient in managing dentinal hypersensitivity. Tal et al. (14) demonstrated that desensitizing effect of fluoride is related to precipitated fluoride compounds mechanically blocking dentinal tubules. Also topical NaF applications create a barrier on the tooth surface, blocking dentinal tubules and reducing hypersensitivity (15).

An essential amino acid, arginine was first isolated from a lupin seedling extract in 1886 by the Swiss chemist Ernst Schultze and has been investigated as arginine bicarbonate together with calcium carbonate for its ability to occlude dentin tubules and reduce pain from DH (16). The Colgate-Palmolive Company develops the new Pro-Argin technology for the treatment of dentin hypersensitivity for this purpose. It is available as office desensitizing paste and toothpaste. The Pro-Argin technology consists of 8% Arginine-CaCO3, and the content of calcium in the form of an insoluble calcium carbonate (17). Since the arginine and calcium carbonate can occlude the exposed dentin tubules and preventing the movement of dentinal fluid in the tubules.

The objective of this study was to determine of dentine hypersensitivity, which occurs following periodontal therapy, and to compare the efficacy of 1.23% NaF-gel and %8 Arginine-CaCO3 after direct topical applications at office. In addition, an in vitro scanning electron microscopy (SEM) study to compare the effectiveness of these agents in occluding dentinal tubules was carried out.

## Material and Methods

The clinical study was a single-center, double-blind, randomized design. The study protocol has been reviewed and approved by the Ethical Board of Gazi University School of Medicine, and volunteers were asked to give an informed written consent to participate, after a thorough explanation of the safety and potential efficacy of 8% Arginine-CaCO3 paste, and the probability of receiving 8% Arginine-CaCO3 paste or 1.23% NaF-gel. The study was performed in accordance with the Helsinki Declaration of 1975, as revised in Tokyo 2004.

-Patient selection

Thirty-six female patients (mean age 33.2±4.5 years, range 27-43 years) with a history of DH at least one tooth were selected from the Department of Periodontics of Gazi University, Ankara.

Inclusion criteria were at least one vital tooth with hypersensitivity on facial surfaces to thermal, mechanical, tactile stimuli. Individuals were systemically healthy and non-smokers. They had DH following periodontal therapy and did not use other hypersensitivity methods. Subjects were not treated periodontally and didn’t use antibiotics within the last 6 months.

Exclusion criteria for teeth; 1- with caries; 2- with crowned and prosthetic restoration; 3-with any other painful pathology; 4- restored less than three months; 5- with any restorations into the test area. Qualified patients were enrolled study from January 2010 to December 2010.

-Study design

The design of this study was double-blinded. Patients were allocated to groups according to toss a coin by single examiner (Ö.E) who applied the desensitizing agents and blinded to all measurements. The 1.23% NaF-gel and 8% Arginine-CaCO3 containing sealer had the same appearance. No one, including the other examiner (A.U), operator collecting clinical measurements, and the patients aware of the type of desensitizing agent to which the patients was assigned.

**Figure 1:**
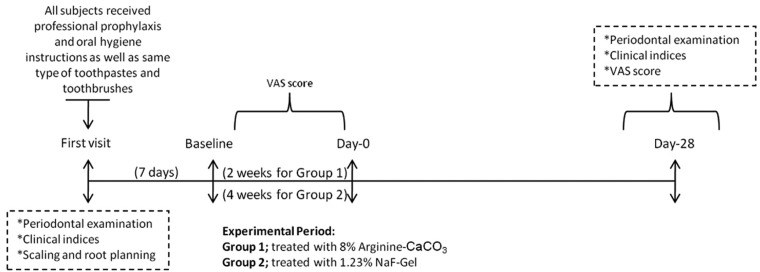
The study protocol performed in the study.

Study protocol is summarized at figure 1. At their first visits, prior to professional prophylaxis, clinical periodontal parameters were recorded. Before the experimental phase, each subject received professional prophylaxis and was given detailed oral hygiene instructions. Subjects were provided with a kit containing same type of standard toothpastes and toothbrushes (Colgate, Istanbul, Turkey) for oral hygiene. Subjects maintained self-performed oral hygiene measures between visits.

The periodontal therapy including supra- and sub-gingival scaling, root planning and polishing (SRP) were performed. SRP process implemented 20 strokes for each tooth surface (18). Following standard periodontal therapy, hypersensitive teeth were identified by the patient and verified by the light stroke of a dental explorer along the cervical area of all teeth present. The tooth form maxillary or mandibular arches of each subject were selected in this study.

To assess tooth sensitivity, tactile test, a controlled air stimulus (evaporative stimulus) and cold water (thermal stimulus) were used. For all stimuli tests the response of patients were recorded and also the subjects placed a mark on a 10 cm–long line on the Visual Analog Score (VAS) (19) that was labeled from “no pain” (0) to “intolerable pain” (10). Tactile test, air blast test and cold water test were performed by single researcher (A.U) and reevaluated at three time points: end of the periodontal therapy (baseline); immediately after applications of desensitizing agents at Day-0; four weeks after applications of desensitizing agents.

1) Tactile test: A sharp dental explorer was passed lightly across the affected mostly at cervical area of the tooth, perpendicular to the long axis of the tooth. The test was repeated three times before a score.

2) Air blast test: A blast of air from a dental syringe at 60 pound/inch 2 pressure was directed onto the affected area of the tooth for 1 second from a distance of 10 mm (measured by taping a scale to the three-way syringe); the adjacent teeth were protected using cotton rolls.

3) Furthermore, 10 µl of ice-cold water applied to the exposed dentin surface while neighboring teeth were isolated during testing using cotton rolls. Sensitivity was measured using VAS score. A period of at least 5 minutes was allowed between the two stimuli on each tooth.

-Application of Desensitizing Agents

A total of 71 teeth with hypersensitivity were included in this study after the stimuli test. Patients randomly divided into two groups. A week after scaling and root planning, each tooth treated with 1.23% NaF-gel or 8% Arginine-CaCO3 by a single researcher (Ö.E). Patients and other researchers did not know which gel had been delivered.

The group 1 was included 34 teeth from eighteen patients treated with 8% Arginine-CaCO3 (Colgate Sensitive Pro-Relief ™, NY, USA).8% Arginine-CaCO3 containing sealer was applied to the teeth with a micromotor and round brush, for 3 second per teeth, according to manufacturer’s instructions.One week later, tooth received second application.

The group 2 was included 37 teeth from eighteen patients treated with 1.23% NaF-gel (Topex Topical Acidulated Phosphated Fluoride Gel, Sultan Inc., Englewood, NJ, USA). All teeth were isolated with cotton rolls before the 1.23 % NaF-gel application and the gel was applied for 30 seconds per teeth of group 1 with a cotton pellet once a week for 4 weeks.

Patients were controlled one month after applications and all measurements were repeated.

-Clinical measurements

The clinical examination included plaque index (PI) (20), gingival index (GI) (21), pocket probing depth (PPD), clinical attach-ment level (CAL), gingival recession width (GRW), gingival recession length (GRL) and mobility were recorded from all subjects at their first visit. A single examiner (A.U), blinded to the desensitizing agents applied, performed all measurements. The meas-urements were recorded at 6 sites per tooth (mesio-vestibular, mid-vestibular, disto-vestibular, mesio-lingual, mid-lingual, disto-lingual) using the Williams periodontal probe calibrated in millimeters (Nordent Manufacturing Inc., Elk Grove Village, IL, USA).

-In vitro study:

10 freshly extracted teeth (5 teeth: 1.23 % Sodium Fluoride group and 5 teeth: 8% Arginine-CaCO3 group) were prepared and treated as mentioned above and subjected to scanning electron microscopy to assess the tubule occlusion like the clinical applications. Extracted, caries-free single rooted teeth were fixed in 3% glutaraldehyde buffer for 1 week and teeth were cleaned for removing of organic debris.

1.23% NaF-gel was applied with cotton rolls to the buccal surfaces of first five teeth %8 Arginine-CaCO3 paste was applied manually to the buccal surfaces of other five teeth. The buccal surfaces of the teeth were used as test groups and the palatinal / lingual surfaces of the teeth were used as control groups.The teeth were embedded into the acrylic blocks and 1mm discs were obtained from the region above the cemento- enamel junction with a0.2 mm megatome knife. Test and control regions are marked. The discs were etched with %3 phosphoric acid for 1 minute and afterwards they were cleaned with distilled water (4,6).

-The Scanning Electron Microscopy

Test and control dentine specimens were mounted flat for surface views. They were than sputter coated with gold (Polaron sc 502 Sputter Coater). Discs were examined in (SEM JEDL JSM – 6060 LV, Japan). Micrographs were taken from selected buccal and lingual aspects of each disc at varying magnifications (x 75,x 1000, x 2500, x 5000). The digitized image of the etched dentine surface with open dentine tubules after applications was calibrated using 5μm bar. Images measured by using image analysis software (Leica Q-Win V-3 Plus, Leica Microsystems, Germany) and data stored in Excel.

-Statistical Analysis

Statistical analysis was performed using a Statistical Package for Social Sciences 16 software (SPSS Inc., Chicago, IL, USA). Differences between before and after VAS scores within each group were analyzed by Wilcoxon Signed Ranks test and comparisons between groups were analyzed by Mann-Whitney U test. Correlation was evaluated by using Spearman’s Rho correlation coefficient. Non-parametric tests were used because of assumption of normality and homogeneity of variance is not supported. Significance level was taken as 0.05.

## Results

-Clinical Results

Thirty-six subjects complied with the protocol and completed the clinical study from September 2009 to August 2010. A summary of the study population is presented in  Table 1.

**Table 1:**
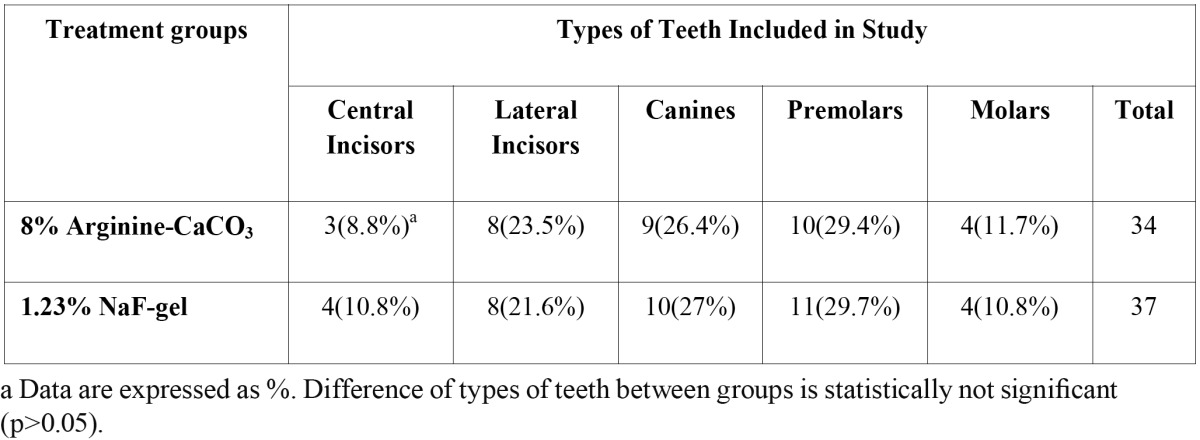
Summary of the study population.

Throughout the study, there were no adverse effects on the soft or hard tissues of the oral cavity, which were observed by the examiner or reported by the subjects when questioned. Types of teeth included in the study are shown in  Table 1. The canines and premolars were most affected tooth in both groups, followed by the lateral and central incisors, while molars were the least affected.

Throughout the study, plaque accumulation was minimal and gingival health was excellent in all subjects. There were no statistically significant differences on clinical parameters between groups at first visit (p>0.05) ( Table 2).

**Table 2:**
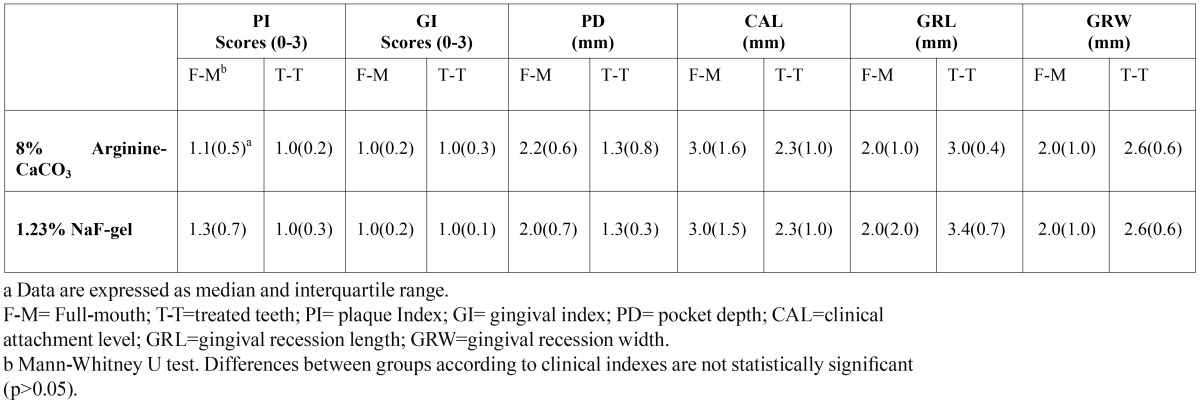
The comparison of clinical indices between groups at baseline.

Mean VAS scores for tactile, air and thermal stimulus of 1.23% NaF-gel group and 8% Arginine-CaCO3group at baseline, Day-0 and Day-28 are shown in  Table 3. VAS scores for 3 stimuli of all two groups were not statistically different from each other at baseline (p>0.05).

**Table 3:**
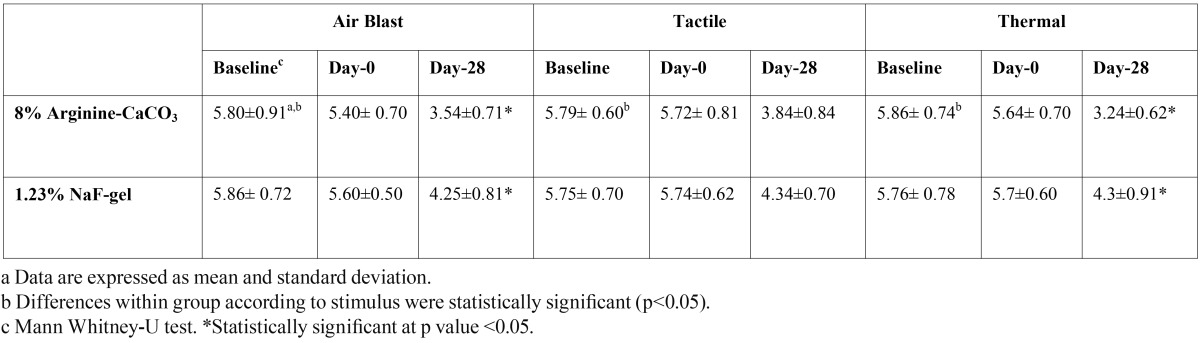
Sensitivity Scores to Three Stimuli for Both Treatment Group at All Time Points.

The VAS score for three stimuli were not statistically different among the groups at baseline and Day-0 (p>0.05). The 8% Argin-ine-CaCO3 group was found to be better in reducing VAS score for air-blast stimuli, tactile stimuli and thermal stimuli compared to the 1.23% NaF-gel group. The changes of air-blast stimuli and thermal stimuli were highly significant in the Arginine group at Day-28 than NaF-gel group (p<0.001). The 8% Arginine-CaCO3 group was more effective for tactile stimuli than NaF-gel group at Day-28, however the differences between groups was not statistically significant (p>0.05). All teeth with DH in this study had some degree of gingival recession. Significant correlation was found between gingival recession length and VAS score at baseline and Day-0 in 8% Arginine-CaCO3 group (ρ=0,692) and 1.23% NaFgroup (ρ=0,572) (p<0.05).

Group 1. The differences of VAS score for air-blast stimuli, tactile stimuli and thermal stimuli were significant at baseline to Day-28 (p<0.05). There was greater reduction in sensitivity score for thermal stimuli at Day-28, following air-blast and tactile stimuli respectively. The VAS score was statistically significant at time interval during experiment for three stimuli.

Group 2. The changes in VAS score of air-blast stimuli, tactile stimuli and thermal stimuli at 1 month were decreased to compare with baseline, decreasing was statistically significant (p<0.05). At Day-28, the VAS scores for air-blast, tactile and thermal stimuli were lower than Day-0. These differences were statistically significant.

-In vitro Results

**Figure 2:**
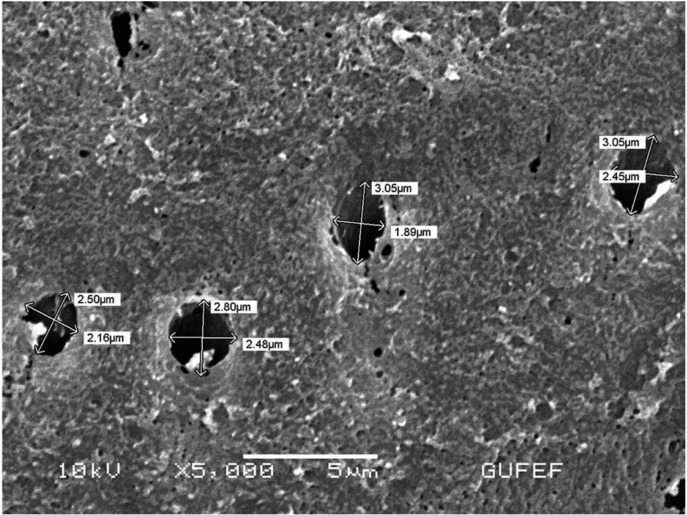
The SEM micrograph of an etched dentine surface treated with 8% Arginine-CaCO3. Particles of various sizes can be seen on the surface and, on occasion, within the tubule opening. μbar represents 5μm (x5000).

**Figure 3:**
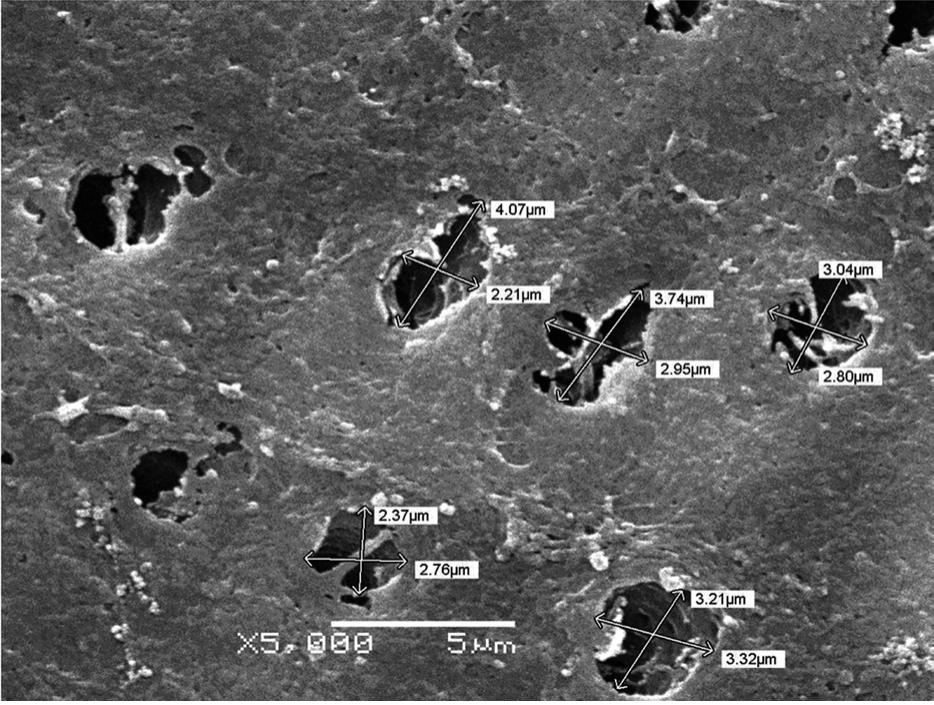
The SEM micrograph of an etched dentine surface treated with 1.23% NaF-gel. There are some product particles on the dentine surface, and at the tubule opening. μbar represents 5μm (x5000).

The scanning electron microscopy results showed effective tubule occlusion in tooth specimen treated with 8% Arginine-CaCO3 ( Table 4) (Fig. 2) than 1.23% NaF-gel (Fig. 3). Following 8% Arginine-CaCO3 and 1.23% NaF-gel applications, the differences of tubule occlusion were statistically significant between groups.

**Table 4:**
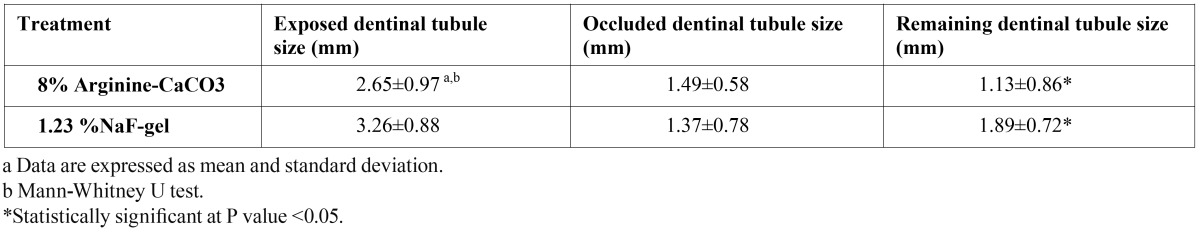
The results of scanning electron microscopy analysis.

## Discussion

The aim of the study was to evaluate the short-term clinical efficacy of a product containing 8% Arginine-CaCO3 and 1.23% NaF-gel which is used for prevention of dentine hypersensitivity that usually occurs following periodontal treatment. Several studies have shown that periodontal treatment frequently is associated with DH (22,23). It has been reported that periodontal therapy appears to be a significant cause of dentin hypersensitivity (24,25). After periodontal therapy, reduction of gingival pro-tective barrier may result from excision of tissue that exposes the root surfaces, while SRP may remove 20 to 50 micrometers of cementum and expose the dentinal tubules to external stimuli (26). Tammaro et al. (24) observed significant change in dentin hypersensitivity after SRP. Von Troil et al. (25) found that dentin hypersensitivity occurred in approximately one-half patients after they underwent SRP. The finding of this study about the increase of DH after periodontal therapy is consistent with previous studies.

Dentin sensitivity may differ according to different stimuli, and it is recommended that at least two hydrodynamic stimuli be used in the clinical trial. The chemical, mechanical and thermal stimuli to exposed dentin surfaces cause patients to feel pain in teeth (9). Despite only air blast test was applied in many studies, the use of tactile and cold stimulus is also recommended, because, some patients may not experience sensitivity when only air blast stimuli is used (3). Therefore in our study, all three methods were performed, and the 5-minute gap was allowed between two stimuli to minimize interactions. We used the VAS in this study to evaluate hypersensitivity of the patients asthe scale is simple to administer and it has been used to evaluate dentin hypersensitivity, previously. Several investigators have been demonstrated the validity and reliability of the VAS for both experimental and clinical pain (22,24,27). The VAS also appears to be more sensitive in discriminating between various treatments and changes in pain intensity (27,28).

Sodium fluoride was recommended as a desensitizing agent to treat the sensitivity of dentin. The 1.23% NaF-gel was used as a positive control in our study because it has proved to be clinically efficient in the treatment of DH (11,29). Previous studies reported that NaF forms an effective barrier and results in desensitization of dentin (29,30). Many treatment methods have been used but not definitely preferred or highly believable as desensitizing agents.

8%Arginine-CaCO3 products seal exposed dentinal tubules and blocking external stimuli and reduce pain sensation. A recent clinical trial showed that 8% Arginine-CaCO3 can effectively reduce dentin hypersensitivity (5). In the present study we want to investigate the effect of 8% Arginine-CaCO3 for the treatment of dentine sensitivity compared to NaF. According to clinical and in vitro results in our study, Arginine-CaCO3 was better than NaF in both tubule occlusion and decrease of dentine hypersensitivity. The changes of air-blast stimuli and thermal stimuli were highly significant in the Arginine-CaCO3 group at 1 month than NaF-gel group (p<0.001). However %8 Arginine- CaCO3 group was found also to be better in reducing VAS score for air-blast and thermal stimuli compared to the NaF-gel group at Day-0. But it was not statistically significant (p>0.05). The VAS scores for air-blast and thermal test reduced in two groups, but there was a significantly reduction in Arginine-CaCO3 group (p<0.001). The 1.23% NaF-gel was used as a positive control in our study because it has proved to be clinically efficient in the treatment of DH. It has been reported that single-dose application of NaF or other agents is not enough for treatment of DH and, repeated applications are necessary in practice (9). Even though other studies have reported that a single application of topical fluoride decreases hypersensitivity for 24 week (10).

Previous clinical studies have shown that the evaluation period of desensitizing agents must be a minimum of 4 or better 8 weeks (1,4,9,10). In our study, patients evaluated for a month as, it has been reported that arginine-containing agent blocks the dentin tubules immediately after the application and maintains this effect for 28 days.

Incisors and premolars are more meticulously brushed teeth, DH and gingival recession is more common in these tooth (2,3). Further, buccal surfaces were more affected compared to palatal/ labial surfaces (2,18), depending on brush trauma, and use of toothpaste, containing more abrasive particles. It has been reported that perceived sensitivity due to attachment loss is higher than due to gingival recession (3). In this study, we found out that canine and premolars were the most commonly affected teeth. This result is confirmed to previous studies.

Sensitive teeth associated with periodontal attachment loss and gingival recession. If the cemento-enamel junction exposed to the oral environment, symptom of hypersensitivity induce easily (3). Authors suggested that gingival recession is one of the important causes of dentin exposure, and major predisposing factor for DH (7,8,13). The results of present study showed that a gingival recession is highly correlated with the increase of DH.

As it is not sufficient to evaluate sensitivity and pain only with clinical data (4), in vitro studies are highly needed to investigate the tubule occlusion. When discs were treated with 8% Arginine-CaCO3, the results produced highly significant changes in all test materials (p<0.05). The SEM results have confirmed that Arginine-CaCO3is statistically more effective than NaF-gel in tubule occlusion. The SEM photographs of the tooth specimens treated with Arginine-CaCO3 were in agreement with the clinical results.

Our study indicated that periodontal treatment was one of the etiological factors of DH. The results of present study showed that application of both agents was observed to reduce sensitivity. We found statistically significant differences between the desensitizing efficacy of 8% Arginine-CaCO3 and that of the 1.23% NaF-gel. The 8% Arginine-CaCO3 might be an effective agent in the prevention of dentinal hypersensitivity caused by scaling and root planning procedures. There are needed more long-term studies with a larger sample size to determine the efficacy of desensitizing treatments.
